# Glossiness and Perishable Food Quality: Visual Freshness Judgment of Fish Eyes Based on Luminance Distribution

**DOI:** 10.1371/journal.pone.0058994

**Published:** 2013-03-11

**Authors:** Takuma Murakoshi, Tomohiro Masuda, Ken Utsumi, Kazuo Tsubota, Yuji Wada

**Affiliations:** 1 Food Function Division, National Food Research Institute, National Agriculture and Food Research Organization, Ibaraki, Japan; 2 Department of Ophthalmology, School of Medicine, Keio University, Tokyo, Japan; University of California San Diego, United States of America

## Abstract

**Background:**

Previous studies have reported the effects of statistics of luminance distribution on visual freshness perception using pictures which included the degradation process of food samples. However, these studies did not examine the effect of individual differences between the same kinds of food. Here we elucidate whether luminance distribution would continue to have a significant effect on visual freshness perception even if visual stimuli included individual differences in addition to the degradation process of foods.

**Methodology/principal findings:**

We took pictures of the degradation of three fishes over 3.29 hours in a controlled environment, then cropped square patches of their eyes from the original images as visual stimuli. Eleven participants performed paired comparison tests judging the visual freshness of the fish eyes at three points of degradation. Perceived freshness scores (PFS) were calculated using the Bradley-Terry Model for each image. The ANOVA revealed that the PFS for each fish decreased as the degradation time increased; however, the differences in the PFS between individual fish was larger for the shorter degradation time, and smaller for the longer degradation time. A multiple linear regression analysis was conducted in order to determine the relative importance of the statistics of luminance distribution of the stimulus images in predicting PFS. The results show that standard deviation and skewness in luminance distribution have a significant influence on PFS.

**Conclusions/significance:**

These results show that even if foodstuffs contain individual differences, visual freshness perception and changes in luminance distribution correlate with degradation time.

## Introduction

Every day, consumers make choices about food quality, choosing from among many samples in the market. Freshness is one of the most important factors in fish quality [1], [2]. A number of measures, including biochemical, chemical, physical and microbiological techniques, have been developed to measure freshness in food [3], [4]. However, consumers usually cannot use these measures for freshness in the marketplace, but must rely on sensory cues such as appearance, texture, sound, taste and smell. Previous research has indicated that the results of these sensory assessments of freshness are highly correlated with freshness scores from chemical parameters, and provide a simple and low cost method of judging the freshness of food [5], [6]. In particular, visual properties can include very rich information for food freshness perception [7]–[10]. For instance, Péneau et al. [9] found that shininess on the surface of food contributes to the perceived freshness of strawberries and carrots.

In the field of vision science, material perception studies focus on the analysis of visual cues that may underlie our ability to discriminate between the different properties of an object. For example, material perception has been studied for stucco-like surfaces [11] and Lambertian surfaces [12]. Motoyoshi et al. [11] revealed that glossiness or the material perception of visual objects varied with image statistics on the surface of objects. In their experiments, the appearance of a visual object was perceived to be glossier as the skewness of the luminance histogram increased.

These statistics of luminance distribution in images are also determining factors for the perceived freshness of food [7], [8], [10]. Wada and colleagues [7], [8], [10] found a correlation between perceived freshness and the values of luminance distributions and spatial frequency in images of individual fresh foodstuffs (cabbages and strawberries). However, stimuli used in these studies were patches from photographs of one cabbage leaf or one strawberry, and did not include individual differences among the same foods. For example, when consumers buy an apple in a grocery store, they must choose from many individual apples with various optical differences. Consumers perceive the differences among them and choose the one which looks the best. Of course, the luminance distribution of each image of an individual item would also include these differences. Thus, previous research could not show whether image statistics such as luminance distribution can predict the degradation of food among samples including items with individual differences. If image statistics were important factors in the perception of the freshness of foodstuffs with individual differences, this study might provide a powerful contribution to the development of a simple and low cost freshness estimation system using image statistics. In this study, we investigated whether or not statistics in luminance distribution are among the determining factors in the visual freshness perception of fresh foods even when individual differences are included in stimulus images. We further investigated to determine which parameters were involved and how they affected the perception of freshness. In order to simulate the daily observation and choice of fresh foodstuffs, we used the paired comparison method to measure perceived freshness. The paired comparison method has been used for measuring food preference [13] and visual preference for products [14], and is useful in the determination of preferences. This method allows us to measure freshness perception in comparisons of fresh foodstuffs in a manner that is close to a typical purchase situation. In this study, we chose fish eyes as the stimuli, because the glossiness of a fish's eye plays an important role in assessing the freshness of the fish. After death, fish become dry and wrinkled due to loss of surface moisture, and this initially occurs in the eye. In relation to the glossiness of the fish eye and the freshness of the fish, it has long been known that there is a strong correlation between the degree of fish freshness and the eye fluid refractive index (RI) value of the fish [15], [16]. The eye fluid of a fresh fish is bright and transparent. This brightness is lost with time due to drying. Therefore, the light refraction properties of the eye fluid can be used as a quality criterion to assess the freshness of fish [15]. However, refractometers are necessary to measure RI whereas consumers evaluate the glossiness of a fish's eye without machines when they purchase food. In addition, in a previous study on human dry-eye patients, Goto et al. [17] showed that tears contribute not only to ocular surface wetness but also to the extent of light reflection. This finding suggests that the intensity of corneal light reflection reflects tear volume and ocular surface wetness. Thus, we can assume that fish eyes lose wetness and light reflection as the degradation time increases, and that this should be accompanied by a change in the luminance distribution of the fish eye image.

## Materials and Methods

### Ethics statement

We used three fishes (horse mackerel; *Trachurus japonicus*) that we randomly selected from a local market on April 21, 2011. The research followed the tenets of the Declaration of Helsinki. Written informed consent was obtained after a complete explanation of the study. The study was approved by the institutional ethics committee of the National Food Research Institute.

### Participants

Eleven volunteers participated in the experiment (mean age = 31.45 *SD* = 8.19). All of the participants reported normal or corrected-to-normal visual acuity, normal color vision, and no history of neurological problems. No experts on cooking, trading, fish farming, or the sensory evaluation of food were included. We conducted no specific training for participants.

### Apparatus

The visual stimuli were presented on a 22-in CRT monitor (Iiyama HM204DA) using ViSaGe (Cambridge Research Systems Co. Ltd.).

### Sample

We used three fishes (horse mackerel; *Trachurus japonicus*) that we randomly selected from a local market on April 21, 2011. The photographs used in the experiments were taken on the date of purchase and the day after.

### Stimulus Images

**Figure 1:**
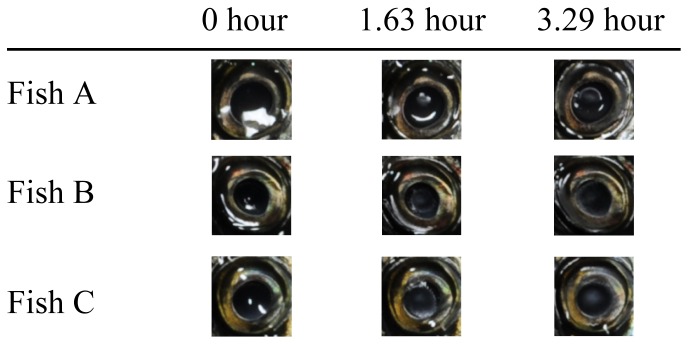
Stimulus images used in the experiment. Stimulus images were patches of 128×128 pixels (4.7×4.7 degrees of arc) of the eyes of three individual fishes from photographs of the freshness degradation process taken at 0, 1.63 and 3.29 hours.

The images used in the experiment were taken in a dark room in which the humidity and the temperature were kept at about 23% and 29.0°C, respectively. A digital camera (Nikon D3) was set up using a tripod in a box designed for taking photographs (D' CUBE J; 116×100×100 cm). Illumination was achieved with two floor lamps with a color temperature of 5400K. We took 4256×2832 pixel photos automatically every 2.5 min for 197.5 min (3.29 hours). As stimulus images, we used 128×128 pixel (4.7×4.7 degrees of arc) patches of the eyes of the three individual fishes from the photographs of the freshness degradation process taken at 0, 1.63 and 3.29 hours (see Fig. 1). The purpose of this selection was to investigate whether observers would perceive freshness as a negative function of degradation time. Table 1 shows the statistics of luminance distribution for nine stimulus images (three fishes at three degradation times). These images involve not only the difference between the degradation times, but also individual differences including different positions relative to the camera and illumination.

**Table 1:** Statistics of luminance distribution in each stimulus image.

	Fish A			Fish B			Fish C		
Time (hour)	0	1.63	3.29	0	1.63	3.29	0	1.63	3.29
*Average (cd/m^2^)*	66.24	62.73	67.03	60.93	65.72	73.35	47.44	54.88	53.21
*SD (cd/m^2^)*	64.69	51.83	49.66	53.01	49.83	49.28	42.47	40.46	41.97
*Skewness*	1.49	1.44	1.50	1.21	0.87	0.80	1.67	1.06	0.97
*Kurtosis*	4.46	4.61	5.38	3.83	2.99	2.99	5.41	3.32	3.10

### Procedure

The participants' heads were fixed to a chin rest about 57 cm from the screen. Participants binocularly observed the presented stimuli in a dark room after a dark adaptation period of 10 min. Two stimulus images, which were positioned side-by-side 7 degrees apart in visual angle, were presented on the screen. Participants were required to report which of the two fish in the stimulus images they perceived to be fresher by pressing one of two keys, for a total of 720 trials (comparison of each of the 9 images with each other, yielding 9×8 = 72 comparisons×10 times). For each of the 72 pairs of eye presentations, each of the eyes was presented on the right 50% of the time and on the left the other 50%.

### Analysis of data

For each participant, we calculated the perceived freshness score (PFS) for each image using the Bradley-Terry model [18] for comparison on the uni-dimensional scaling. The PFS were analyzed by analysis of variance (ANOVA) with within-subject factors of individual differences between each fish and degradation time. *P*<.05 was considered statistically significant. In order to clarify the relationship between the statistics of luminance distribution (average luminance, luminance standard deviation, luminance skewness, luminance kurtosis) and the PFS, a multiple linear regression analysis was also conducted.

## Results

### Yardstick of perceived freshness scores

**Figure 2:**
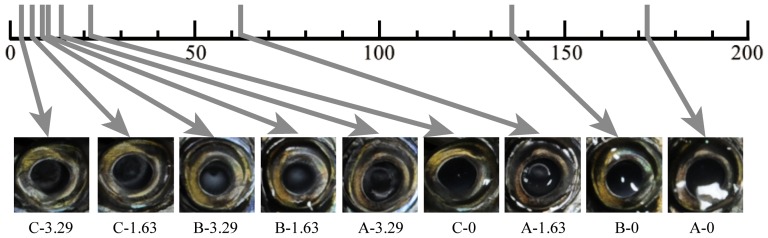
Yardstick which lines up the perceived freshness scores of each image according to their scores on a one-dimensional line. Each vertical gray line on the yardstick indicates the perceived freshness score (PFS) of each image. Distance from the left edge of the scale to each vertical gray line depicts the score size of each image: an image indicated by a vertical gray line positioned nearer the right side was perceived as fresher than that nearer the left side. Labels under the images indicate the individual identification index and degradation time of each image.

The PFS were calculated using the Bradley-Terry Model for each image from the frequency with which one fish was perceived to be fresher than another. Figure 2 shows the yardstick on which one-dimensional lines of PFS for each image are plotted according to their scores. A χ^2^ test revealed agreement between participants' responses for perceived freshness (χ^2^ = 297.11, *p*<.01). As shown in Figure 2, although each individual fish has different PFS values, an image with a shorter degradation time was perceived as fresher than one with a longer degradation time within each individual fish. In addition, the differences between the images were large when PFSs were high, whereas they were small when the scores were low.

### ANOVA of perceived freshness scores

**Figure 3:**
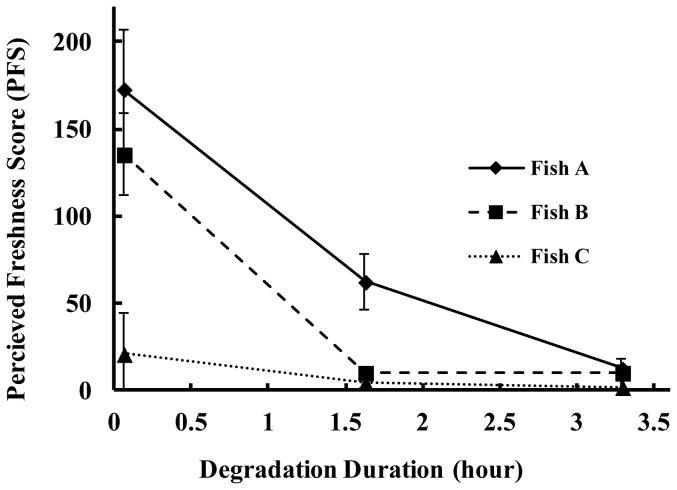
Perceived freshness score (PFS) in each stimulus image as a function of degradation time. The x-axis indicates the degradation time (hour) and the y-axis indicates PFS. Each line represents a function of degradation time of each individual fish. The vertical bars indicate standard error (*SE*).

Figure 3 shows the PFS for each stimulus image as a function of degradation time. The PFS for each fish decreased as the degradation time increased; however, the slopes of these scores differed greatly between each fish. As in Figure 2, the differences between the PFS of each fish were larger at the shorter degradation time, and smaller at the longer degradation time.

The ANOVA identified degradation time as having a significant effect on PFS [*F*(2, 40) = 36.03, *p*<.01]. The effect of individual differences was also significant [*F*(2, 40) = 16.04, *p*<.01]. Further, interaction was observed between the effects of degradation time and individual differences [*F*(4, 40) = 5.27, *p*<.01]. The simple main effects for degradation time and individual differences were examined using a post hoc test, identifying the simple main effects for degradation time in all fishes [*F*(2,9) = 22.20, *p*<.01 for fish A, *F*(2,9) = 12.91, *p*<.01 for fish B, *F*(2,9) = 12.36, *p*<.01 for fish C], and individual differences at 0 and 1.63 hours degradation time [*F*(2,9) = 20.69, *p*<.01 for 0 hr, *F*(2,9) = 6.06, *p*<.05 for 1.63 hr, *F*(2,9) = 1.60, *n.s.*, for 3.29 hr].

### Multiple linear regression analysis on statistics of luminance distribution and perceived freshness scores

**Figure 4:**
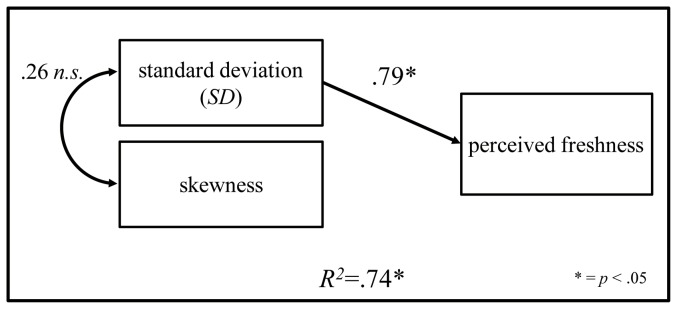
The regression model, which is most appropriate for accounting for PFS computed by multiple linear regression analysis. Two luminance distribution dimensions (standard deviation and skewness) have a significant influence on PFS (*R^2^* = .74, *F* = 8.43, *p*<.05). Only standard deviation significantly and positively influenced PFS (beta = .79, *p*<.05).

**Figure 5:**
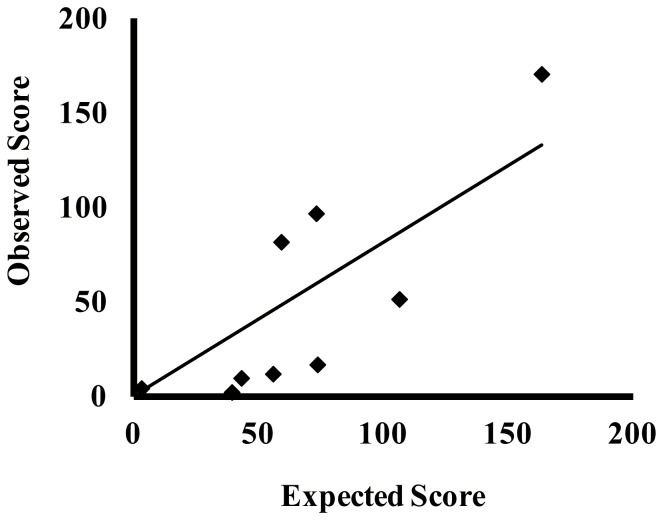
Predicted score from the regression model vs. actual perceived freshness score.

Multiple linear regression analysis was conducted in order to determine the relative importance of the luminance distribution statistics of the stimulus images in predicting PFS. In identifying the significant variables accounting for PFS, it was found that standard deviation and skewness of luminance distribution had a significant influence on PFS. The adjusted *R^2^* of this model is .74, which indicates that 74% of variation in PFS was explained by these two dimensions. The significant F-ratio (*F* = 8.43, *p*<.05) indicates that the results of the regression model were unlikely to have occurred by chance. Thus, the goodness-of-fit of the model is satisfactory. Only standard deviation dimensions significantly and positively influenced PFS. Based on the beta coefficient of each independent variable, it is possible to assess the impact of each variable on PFS. As shown in Figure 4, the standard deviation was an important determinant of PFS; it had the highest standardized coefficient value, .79. Figure 5 shows the relation between the scores predicted using this model, and actual PFS. Actual PFS results were distributed near the line that indicates predicted score, meaning that the model in which standard deviation and skewness of the luminance distribution had a significant influence on PFS predicted actual PFS quite precisely.

## Discussion

We investigated whether luminance distribution would continue to have a significant effect on visual freshness perception even under the condition where visual stimuli included not only the degradation of a food, but also individual differences between foods. The results indicate that even if foodstuffs contain individual differences, the visual freshness perception and changes in luminance distribution still correlate with degradation time.

Results of ANOVA with factors of individual differences and degradation time on PFS revealed that when people assess the freshness of fresh fish from its visual appearance, perceived freshness varies greatly between individual fishes. On the other hand, the effect of degradation over time is consistent within each fish; the PFS of each fish decreased as degradation time increased. These findings suggest that freshness perception from fish eyes is affected by degradation time, and also individual differences between each fish.

The result of multiple linear regression analyses of PFS with statistics of luminance distribution as independent variables shows that PFS are predictable from these statistics in an image. The regression equation implies that the change in the perception of freshness over degradation time in a single fresh foodstuff can be estimated from the variation of luminance distribution in accordance with our previous studies [7], [8], [10]. Moreover, our findings showed that even if some foodstuffs contain individual differences, degradation in the freshness of those foodstuffs can be predicted by their luminance distribution. Multiple linear regression analysis revealed that perceived freshness can be predicted by standard deviation and skewness of luminance distribution; in particular, fresh food is perceived as fresher as standard deviation in its image becomes higher.

The high-adjusted *R^2^*( = .74) of our model indicates that standard deviation and skewness of luminance distribution in an image have an important role in the perception of freshness in food. These statistics may be related to the wetness of the eye. It is indicated that the wetness of the eye affects the luminance distribution in images of the human eye [17], and the light refraction properties of eye fluids in fish change with drying [3]. Fish eyes became dryer with a longer degradation time due to the low humidity (about 23%) in our experiment, so that the luminance distributions of the images of the fish eyes changed according to degradation time. Thus, it can be suggested that changes in standard deviation and skewness of luminance distribution may correlate with wetness on the surface of the eye. This correlation might enable us to perceive the freshness of fish from its image with our visual systems using this information.

Here it should be noted that a skew in luminance distribution does not exactly equate to glossiness. Some recent studies have suggested that the strong correlation between glossiness and histogram is violated if the extremes of distribution do not correspond to the locations of specular highlights of the visual objects [19], [20]. Since the same spatial correspondence may not apply to translucent objects such as fish eyes, there is the possibility that the correlation between luminance distribution change and the perceived freshness of a fish eye might involve not only the surface change of eyes, but also changes in volumetric light-transport properties such as scattering or absorption.

In addition, there were individual differences in the images of the fish in our experiment due to photographic conditions such as the relative position of each fish to the lighting and camera. Previous studies on the relationship between corneal light reflection and ocular surface wetness in humans suggest that the position of the light source, object, and observer may be an important factor that possibly affects the measurement of reflection [17]. Furthermore, the human visual system allows multiple images to be obtained simultaneously or sequentially through binocular disparity and sequentially motion parallax, and such multiple images enhance glossiness perception [21]. Since the effect of motion parallax on glossiness is enhanced by head motion [22], the relationship between the light source, object, and viewpoint cannot be ignored for quality perception. Further research which strictly controls artifacts such as the photographic conditions is necessary in order to elucidate the details of the effect of individual differences between foodstuffs on visual freshness perception.

To conclude, we found that humans perceive the freshness of food as a function of luminance distribution in an image, and this perception correlates with degradation over time even when individual differences between foodstuffs are included. The present findings are potentially useful not only towards determining the mechanism of visual freshness perception, but also towards developing a new technique for the nondestructive evaluation of the freshness of fish or any other fresh food. This approach to revealing the function between human perceptions and optical parameters might allow us to establish a foundation upon which the functions between human perceptions and biochemical parameters can be objectively measured.

Recent studies [23]–[25] using fMRI adaptation and visual object agnosia have suggested that texture and color activate different regions in the human cortex and that glossiness does not depend exclusively upon processing in the same constellation of regions. In addition, Nishio et al. found that particular cortical areas in macaques possess selectivity for glossiness [26]. These findings imply that visual properties such as shape, color, texture and glossiness are separately processed in the brain. Another study using fMRI suggested that the ventral cortex around the fusiform gyrus is related to categorization of materials in humans [27]. The current study, which implies that visual properties such as glossiness might be useful cues for food quality, provides another plausible line of reasoning for the evolutionary advantage of the visual systems to extract glossiness.
